# Differential gene expression profiling linked to tumor progression of splenic marginal zone lymphoma

**DOI:** 10.1038/s41598-017-11389-5

**Published:** 2017-09-08

**Authors:** Tomonori Higuchi, Yumiko Hashida, Ayuko Taniguchi, Mikio Kamioka, Masanori Daibata

**Affiliations:** 1 Department of Microbiology and Infection, Kochi Medical School, Kochi University, Nankoku, Kochi, 783-8505 Japan; 2 Department of Hematology and Respiratory Medicine, Kochi Medical School, Kochi University, Nankoku, Kochi, 783-8505 Japan; 3 Department of Laboratory Medicine, Kochi Medical School, Kochi University, Nankoku, Kochi, 783-8505 Japan

## Abstract

The genetic events that lead to aggressive transformation of cases of splenic marginal zone lymphoma (SMZL) after the chronic clinical stage have not been well understood. We aimed to find candidate genes associated with aggressive features of SMZL. We have successfully established two SMZL cell lines, designated SL-15 and SL-22, derived from the same patient’s tumor clone in chronic and aggressive phases, respectively. Microarray analysis identified cell cycle-associated genes—specifically *PLK1*—as the most significantly upregulated in primary aggressive SMZL cells compared with cells from chronic phase. *EPHA4* and *MS4A1* (*CD20*) were found to be downregulated dramatically. These gene expression patterns were reproduced in both cell lines. Genetic knockdown of *PLK1* resulted in inhibition of cell proliferation and induction of apoptosis in SL-22 cells, which expressed higher levels of *PLK1* than SL-15 cells. SL-22 cells needed higher concentrations of chemical PLK1 inhibitors to achieve greater effects. In addition, we found homozygous deletion of the *MS4A1* gene as a newly identified molecular mechanism of CD20-negative conversion. Our findings are expected to stimulate further studies on whether PLK1 could be a potential therapeutic target for this tumor. Furthermore, cases with CD20-negatively converted lymphomas should be screened for the genomic loss of *MS4A1*.

## Introduction

Splenic marginal zone lymphoma (SMZL), also called splenic lymphoma with villous lymphocytes, is a rare B-cell neoplasm involving the spleen, bone marrow, and usually peripheral blood^1^. Most patients with SMZL show a chronic course with a median survival of around 10 years, whereas in a subset of patients the disease transforms to a more aggressive course with rapidly progressive and treatment-resistant form with increased mortality^2–4^. In the last few years, molecular genetic studies have identified a plethora of somatic mutations in cases of SMZL^5, 6^. The most frequently mutated genes are *KLF2* and *NOTCH2*, with a prevalence of 20–40%^7–9^ and ~10–25%^6, 8, 10^, respectively. Inactivation of *KLF2* and upregulation of *NOTCH2* are involved in the physiological differentiation and proliferation of splenic marginal zone B cells, which might contribute to lymphomagenesis^2^. However, the genetic changes underlying the transformation of SMZL into a high-grade aggressive malignancy remain unknown. Although recognition of the sequential gene expression profiles during progression from chronic to aggressive phases of SMZL is helpful in revealing markers for tumor progression, the rarity of the disease, coupled with a lack of suitable *in vitro* study systems, might have hindered the biologic and genetic investigation of the aggressive transformation of SMZL. This study aimed to identify candidate genes associated with aggressive features of SMZL.

One approach to understand malignant transformation is by comparing gene expression of tumor cells derived from a chronic phase to their evolved malignant counterparts. Cell lines represent invaluable tools for research on rare diseases such as SMZL. Our previous study described an SMZL cell line, SL-15, established form a tumor in a chronic phase^11^. The case had a prolonged chronic clinical course with a good therapeutic response to monotherapy using the anti-CD20 monoclonal antibody rituximab, but later transformed into an aggressive disease. We have again successfully established another cell line, designated SL-22, from the transformed and aggressive tumor in the same patient. Comparison of the primary lymphoma cells as well as their evolved cell lines derived from a single patient with SMZL in two different phases of the disease has provided an opportunity to study sequential gene expression profiles during such transformation. In this study, microarray analysis showed a differential gene expression profile between SMZL cells derived from the chronic and aggressive clinical phases. We raised several therapeutic potential targets especially linked to cell cycle regulation, most notably *PKL1*, for further investigation of the genetic basis of SMZL transformation.

Rituximab-based treatment is a valid therapy for SMZL and is associated with a high overall response rate (~90%), with complete remission in more than half of these responding cases^2, 12^. Although consecutive treatment with rituximab further improves the complete remission rate in patients with SMZL, acquired resistance to this drug has become a considerable problem. Studies have suggested that loss of CD20 expression is a major mechanism in such resistance^13–16^. Epigenetic mechanisms, in part, might contribute to the downregulation of CD20 expression, but the molecular mechanisms are still unclear. One of the major limitations in defining the mechanism of CD20-negative conversion from CD20-positive lymphomas after rituximab use is the lack of a laboratory model by which unlimited supplies of CD20-negative clones derived from CD20-positive cells can be studied repeatedly and extensively. So far, a few CD20-negative cell lines have been established from patients with CD20-positive lymphomas treated with rituximab^17, 18^. However, paired CD20-positive and -negative cell lines derived from the same clones before and after rituximab use, respectively, have been lacking. In this context, our two lymphoma cell lines, SL-15 and SL-22, are valuable for studying the negative conversion of CD20. By utilizing these cell lines, we show here that genomic deletion of the *MS4A1* (*CD20*) gene is another molecular mechanism in the loss of CD20 expression.

## Results

### Comparative characterization of the cell lines

**Figure 1:**
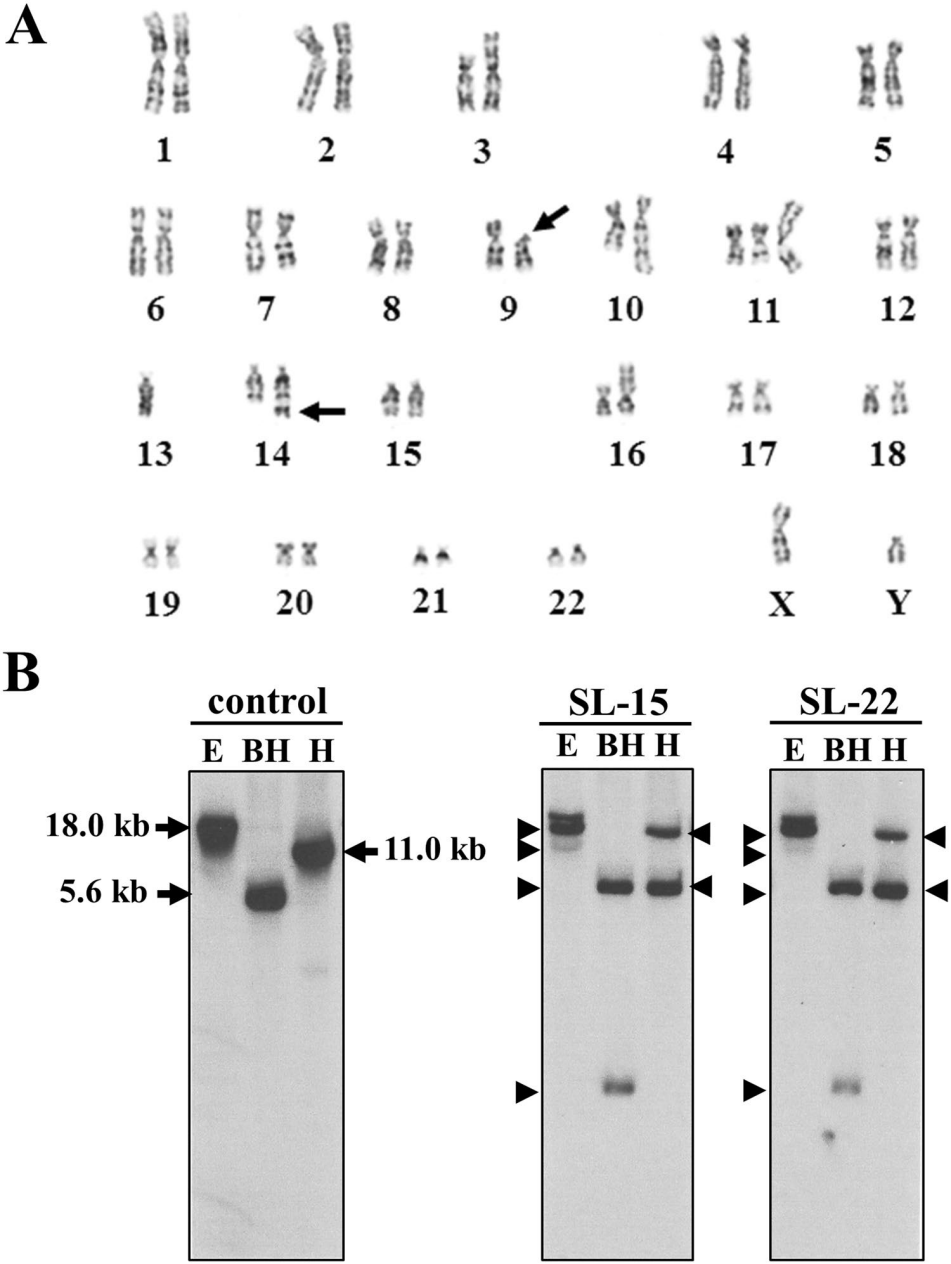
(**A**) Giemsa-banded karyotype of SL-22 cells, showing 47, XY, add(3)(p13), add(3)(p13), t(9;14)(p13;q32), add(10)(q24), add(11)(q21), + add(11). der(11:13)(q10;q10), + 12, and add(16)(p11.2). The karyotype showed a close resemblance to that of SL-15 cells, including a unique chromosomal translocation t(9;14)(p13;q32) (arrows). (**B**) Gene-rearrangement analysis of SL-15 and SL-22 cells. Southern blot analysis revealed rearrangement bands (arrowheads) for the Ig heavy-chain gene. Both cell lines had identical rearrangement bands. Lane E, EcoRI digestion; lane BH, BamHI/HindIII co-digestion; lane H, HindIII digestion.

The Epstein–Barr virus (EBV)-immortalized SL-15 and SL-22 cell lines were established from a single patient with SMZL. The cell surface marker profile of SL-22 cells was similar to that of SL-15 cells, except for being negative for CD20 expression. The karyotype of SL-22 cells was identical to that of the primary SMZL cells at an aggressive phase (Fig. 1A), confirming that these cells were derived from the clone of the patient’s tumor cells. The two cell lines possessed common chromosome aberrations, including a unique t(9;14) chromosomal translocation involving 9q13 and 14q32, where *PAX-5* and the immunoglobulin (Ig) heavy-chain gene are located, respectively^11^, indicating that the SL-15 and SL-22 lines had evolved from the same clone. Southern blot analysis of DNA showed that SL-22 cells exhibited a rearrangement of the Ig heavy-chain gene bands identical to those of SL-15 cells (Fig. 1B), also signifying that the two cell lines were clonally identical. Clearly SL-15 and SL-22 cells are paired SMZL cell lines derived from the same clone.

### Differential gene expression profiles between different clinical periods of SMZL

We compared gene expression profiles of the paired primary SMZL cells derived from the chronic (designated PB-15 cells) and aggressive (PB-22 cells) clinical phases using microarray analysis. A list of the differentially expressed genes was formed under criteria of 2.54-fold upregulation (Z-score > 2) and downregulation (Z-score < –2) in PB-22 cells compared with PB-15 cells (Table 1). A total of 1161 upregulated genes and 1112 downregulated genes were identified and further subjected to gene ontology (GO) analysis using the DAVID analysis. In this, the Functional Annotation Clustering tool identified several significantly upregulated clusters of genes. Annotation cluster 1 showed the highest enrichment score of 10.79 and included genes linked to the cell cycle, cell division, and mitosis (Table 2). Furthermore, pathway analysis (KEGG_PATHWAY) also identified the cell cycle pathway (*P* = 1.16 × 10^−7^) as the most significantly overexpressed one (Table 2). These results indicated that dysregulated expression of genes associated with cell cycle regulation was involved in the aggressive transformation of the disease in our patient.

**Table 1:** Top 30 genes of 1161 upregulated and 1112 downregulated genes in PB-22 cells identified as >2.54-fold (Z-score: >2 or <−2) compared with PB-15 cells.

Upregulated		Downregulated	
Gene symbol^1^	Description	Gene symbol1	Description
TLR5	toll-like receptor 5	**EPHA4**	EPH receptor A4
SYT17	synaptotagmin XVII	MAPT	microtubule-associated protein tau
RGS7	regulator of G-protein signaling 7	GLYCTK	glycerate kinase
FGFRL1	fibroblast growth factor receptor-like 1	RBP7	retinol binding protein 7, cellular
BTNL9	butyrophilin-like 9	ADAMTS9	ADAM metallopeptidase with thrombospondin type 1 motif, 9
NPAS4	neuronal PAS domain protein 4	**MS4A1**	membrane-spanning 4-domains, subfamily A, member 1
GLRA3	glycine receptor, alpha 3	NRN1L	neuritin 1-like
**PLK**	polo-like kinase 1	RGS4	regulator of G-protein signaling 4
DMXL1	Dmx-like 1	TSPAN15	tetraspanin 15
LIFR	leukemia inhibitory factor receptor alpha	SOX5	SRY (sex determining region Y)-box 5
HMBOX1	homeobox containing 1	TNFRSF8	tumor necrosis factor receptor superfamily, member 8
LHFPL2	lipoma HMGIC fusion partner-like 2	**MS4A7**	membrane-spanning 4-domains, subfamily A, member 7
ZNF90	zinc finger protein 90	RAB31	RAB31, member RAS oncogene family
CRYM	crystallin, mu	DENR	density-regulated protein
NXT1	NTF2-like export factor 1	DHRS4	dehydrogenase/reductase (SDR family) member 4
ZNF407	zinc finger protein 407	NOMO1	NODAL modulator 1
CHI3L2	chitinase 3-like 2	MEF2C	myocyte enhancer factor 2 C
ACACB	acetyl-CoA carboxylase beta	SORL1	sortilin-related receptor, L(DLR class) A repeats-containing
TTC39B	tetratricopeptide repeat domain 39B	MACROD2	MACRO domain containing 2
CDCP1	CUB domain containing protein 1	EPDR1	ependymin related protein 1 (zebrafish)
NEBL	nebulette	SDK1	sidekick homolog 1, cell adhesion molecule (chicken)
PLN	phospholamban	TFEC	transcription factor EC
MYBPC2	myosin binding protein C, fast type	HEATR1	HEAT repeat containing 1
TTC3	tetratricopeptide repeat domain 3	MYOM1	myomesin 1
FETUB	fetuin B	OSBPL10	oxysterol binding protein-like 10
CLDN11	claudin 11	DDX60L	DEAD (Asp-Glu-Ala-Asp) box polypeptide 60-like
C9orf93	chromosome 9 open reading frame 93	PTPRU	protein tyrosine phosphatase, receptor type, U
HBD	hemoglobin, delta	ARHGAP18	Rho GTPase activating protein 18
FLJ37543	hypothetical protein FLJ37543	**KLF2**	Kruppel-like factor 2
SPAG1	sperm associated antigen 1	PLEKHA2	pleckstrin homology domain containing, family A (phosphoinositide binding specific) member 2

^1^The underlined genes were investigated further in this study.

**Table 2:** Enriched gene ontology (GO) functions and KEGG pathways for the upregulated and downregulated genes.

Enriched gene ontology functions for the upregulated genes				
Annotation Cluster 1	Enrichment Score: 10.79	Count	*P*-value	Benjamini
Category	Term			
UP_KEYWORDS	Cell cycle ^1^	90	5.97E-17	5.01E-14
UP_KEYWORDS	Cell division ^1^	59	6.10E-13	9.16E-11
UP_KEYWORDS	Mitosis ^1^	46	1.99E-12	2.24E-10
GOTERM_BP_DIRECT	Cell division (GO:0051301)	48	2.95E-08	1.00E-04
GOTERM_BP_DIRECT	Mitotic nuclear division ^1^ (GO:0007067)	36	5.26E-07	4.47E-04
Enriched KEGG pathways for the upregulated genes				
**Category**	**Term**	**Count**	***P*** **-value**	**Benjamini**
KEGG_PATHWAY	Cell cycle ^1^ (hsa04110)	25	1.16.E-07	3.01E-05
KEGG_PATHWAY	p53 signaling pathway (hsa04115)	13	3.85.E-04	4.87E-02
KEGG_PATHWAY	Progesterone-mediated oocyte maturation (hsa04914)	14	1.34.E-03	1.09E-01
KEGG_PATHWAY	Oocyte meiosis (hsa04114)	16	1.39.E-03	8.64E-02
KEGG_PATHWAY	Transcriptional misregulation in cancer (hsa05202)	20	3.61.E-03	1.71E-01
KEGG_PATHWAY	Malaria (hsa05144)	9	6.44.E-03	2.43E-01
KEGG_PATHWAY	Jak-STAT signaling pathway (hsa04630)	17	9.17.E-03	2.89E-01
KEGG_PATHWAY	Biosynthesis of amino acids (hsa01230)	11	9.59.E-03	2.68E-01
**Enriched gene ontology functions for the downregulated genes**				
**Annotation Cluster 1**	**Enrichment Score: 28.63**	**Count**	**P-value**	**Benjamini**
**Category**	**Term**			
INTERPRO	Cadherin, N-terminal (IPR013164)	42	1.05E-35	1.61E-32
UP_SEQ_FEATURE	domain:Cadherin 6	46	1.54E-35	4.90E-32
UP_SEQ_FEATURE	domain:Cadherin 5	49	2.59E-33	4.11E-30
UP_SEQ_FEATURE	domain:Cadherin 2	50	3.39E-32	3.59E-29
UP_SEQ_FEATURE	domain:Cadherin 1	50	3.39E-32	3.59E-29
UP_SEQ_FEATURE	domain:Cadherin 4	49	1.05E-31	8.32E-29
UP_SEQ_FEATURE	domain:Cadherin 3	49	1.05E-31	8.32E-29
INTERPRO	Cadherin (IPR002126)	49	7.89E-30	6.03E-27
INTERPRO	Cadherin conserved site (IPR020894)	48	9.35E-30	4.77E-27
INTERPRO	Cadherin-like (IPR015919)	49	1.98E-29	7.56E-27
SMART	Cadherin repeats (SM00112)	49	6.76E-28	2.18E-25
UP_KEYWORDS	Cell adhesion ^2^	92	1.86E-27	8.65E-25
GOTERM_BP_DIRECT	Homophilic cell adhesion via plasma membrane adhesion molecules (GO:0007156)	53	3.02E-26	1.06E-22
UP_KEYWORDS	Calcium	121	1.12E-22	1.74E-20
GOTERM_MF_DIRECT	Calcium ion binding (GO:0005509)	95	1.07E-14	1.21E-11
**Annotation Cluster 2**	**Enrichment Score: 13.91**	**Count**	**P-value**	**Benjamini**
**Category**	**Term**			
UP_KEYWORDS	Cell membrane ^3^	294	1.20E-24	2.79E-22
GOTERM_CC_DIRECT	plasma membrane (GO:0005886)	351	5.24E-20	2.89E-17
UP_SEQ_FEATURE	glycosylation site:N-linked (GlcNAc)	342	4.47E-18	2.84E-15
UP_KEYWORDS	Glycoprotein	358	4.52E-18	5.26E-16
UP_KEYWORDS	Membrane	520	5.24E-16	5.17E-14
UP_SEQ_FEATURE	topological domain:Extracellular	238	2.10E-14	9.52E-12
GOTERM_CC_DIRECT	integral component of plasma membrane (GO:0005887)	146	1.27E-13	3.50E-11
UP_SEQ_FEATURE	topological domain:Cytoplasmic	273	1.59E-12	5.60E-10
UP_KEYWORDS	Transmembrane	396	1.05E-11	8.15E-10
UP_KEYWORDS	Transmembrane helix	394	1.64E-11	1.09E-09
UP_SEQ_FEATURE	transmembrane region	362	5.12E-11	1.63E-08
GOTERM_CC_DIRECT	integral component of membrane (GO:0016021)	334	4.06E-04	3.66E-02
**Enriched KEGG pathways for the downregulated genes**				
**Category**	**Term**	**Count**	**P-value**	**Benjamini**
KEGG_PATHWAY	Natural killer cell mediated cytotoxicity (hsa04650)	19	1.76.E-04	4.55.E-02
KEGG_PATHWAY	cAMP signaling pathway (hsa04024)	24	8.63.E-04	1.08.E-01
KEGG_PATHWAY	cGMP-PKG signaling pathway (hsa04022)	20	2.82.E-03	2.21.E-01
KEGG_PATHWAY	Hematopoietic cell lineage ^3^ (hsa04640)	13	3.04.E-03	1.83.E-01
KEGG_PATHWAY	Amphetamine addiction (hsa05031)	11	3.95.E-03	1.89.E-01
KEGG_PATHWAY	Tuberculosis (hsa05152)	20	5.74.E-03	2.24.E-01
KEGG_PATHWAY	Cocaine addiction (hsa05030)	9	6.07.E-03	2.06.E-01
KEGG_PATHWAY	Neuroactive ligand-receptor interaction (hsa04080)	27	8.29.E-03	2.41.E-01
KEGG_PATHWAY	Insulin secretion (hsa04911)	12	8.65.E-03	2.26.E-01
KEGG_PATHWAY	Adrenergic signaling in cardiomyocytes (hsa04261)	17	8.91.E-03	2.11.E-01

^1^The underlined terms include *PLK1*. ^2^The underlined term includes *EPHA4*. ^3^The underlined terms include *MS4A1* (*CD20*) and *MS4A7*.

GO analysis was also performed on the downregulated genes. Annotation cluster 1 with the highest enrichment score of 28.63 included genes assigned to GO terms involved in cadherin and cell adhesion (Table 2). The *EPHA4* (*erythroprotein-producing hepatocellular receptor A4*) gene, which was the most strongly downregulated gene (Table 1), belonged to the category of cell adhesion in this cluster. Annotation cluster 2 with an enrichment score of 13.91 contained genes linked to cell membrane functions, including *membrane-spanning 4A* (*MS4A*) family genes such as *MS4A1* (*CD20*) and *MS4A7*. KEGG pathway analysis also identified the *MS4A* family of genes in the inclusive hematopoietic cell lineage pathway as being significantly downregulated (Table 2).

Changes in the expression levels of *KLF*2 and *NOTCH2* were also investigated. Our microarray analysis showed that *KLF2* expression was significantly downregulated in PB-22 cells (18.5-fold lower; Table 1). A higher expression of *NOTCH2* (1.5-fold higher) was observed, although this was not significant.

### Validation of microarray gene expression profiles

**Figure 2:**
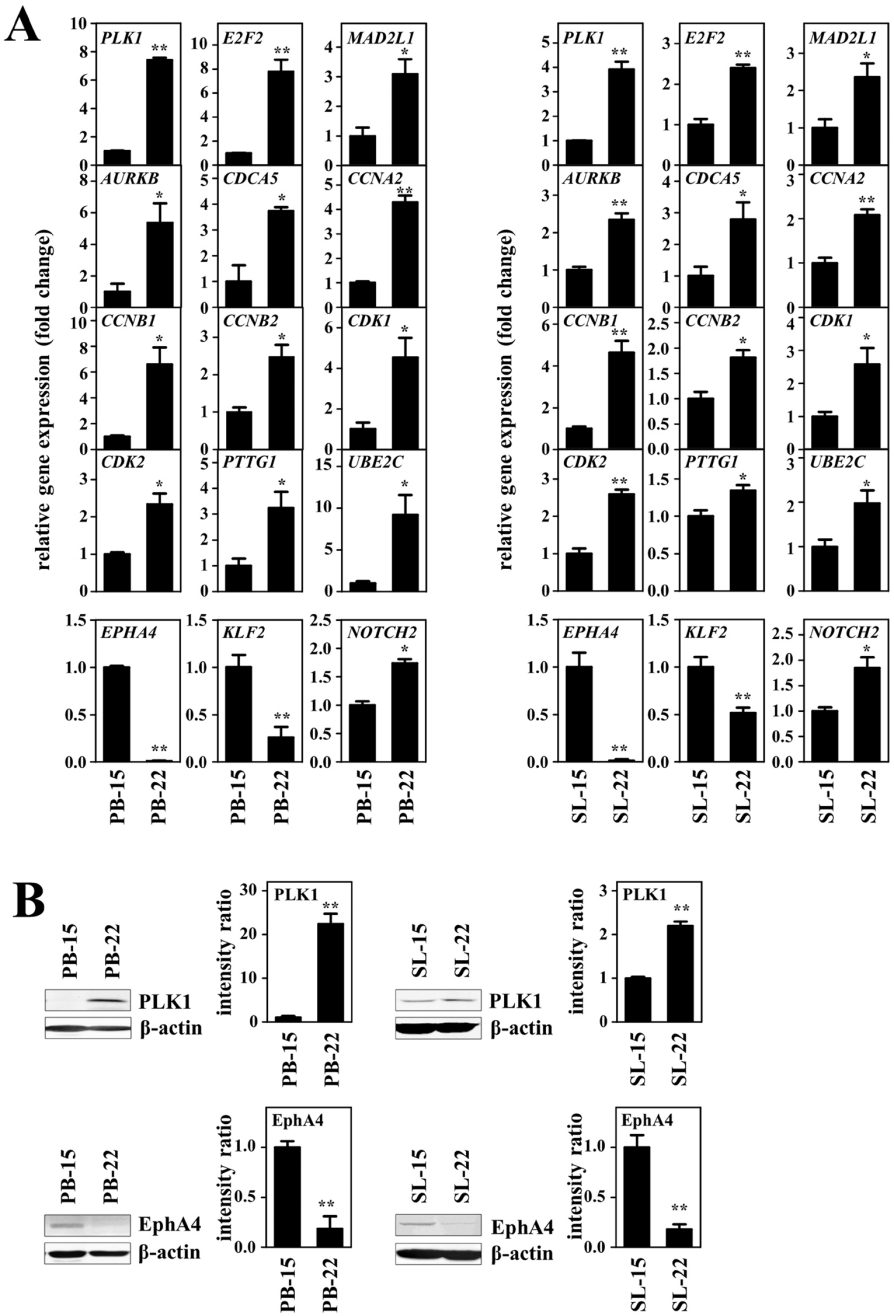
Expression analysis of the target genes in primary SMZL cells (PB-15 and PB-22 cells) and their evolved cell lines (SL-15 and SL-22 cells). (**A**) Analysis of mRNA expression. Differential expressions of genes related to the cell cycle, which showed the highest enrichment score by microarray analysis, were validated by RT–qPCR. Expression levels of *EPHA4*, the most downregulated gene shown by microarray analysis, *KLF2* and *NOTCH2* were also analyzed. Ratios of the expression levels in PB-15 versus PB-22 cells (left panel) and ratios of SL-15 versus SL-22 cells (right panel) are plotted. Data are shown as the mean ± standard deviation (SD) of three independent experiments. (**B**) Analysis of protein expression. Immunoblotting analysis showed upregulation of PLK1 and downregulation of EphA4 in PB-22 (left panel) and SL-22 cells (right panel) compared with PB-15 and SL-15 cells, respectively. Intensities of the bands obtained by immunoblotting were quantified and normalized to the levels of β-actin. The relative amounts of PLK1 and EphA4 in PB-22 cells and SL-22 cells were also normalized to the level (value = 1) for PB-15 cells and SL-15 cells. Data are shown as the mean ± SEM of the three separate experiments. Significant expression differences are shown as *P < 0.05; **P < 0.01. The full-length blots are presented in Supplementary Fig. S3.

The differentially upregulated genes in the cell cycle pathway were validated by real-time quantitative reverse-transcription polymerase chain reaction (RT–qPCR) on primary lymphoma cells (PB-22 versus PB-15 cells) and their evolved cell lines (SL-22 versus SL-15 cells) in three separate experiments. The cell cycle pathway included genes regulating cell proliferation and mitosis. The selected genes were *PLK1*, *E2F2*, *MAD2L1*, *AURKB*, *CDCA5*, *CCNA2*, *CCNB1*, *CCNB2*, *CDK1*, *CDK2*, *PTTG1*, and *UBE2C*. The differential expression patterns were confirmed for all genes in both the primary tumors and cell lines (Fig. 2A). Among the cell cycle related-genes upregulated, *PLK1* showed the greatest difference in expression, as demonstrated by both microarray analysis and RT–qPCR. Immunoblot analysis also validated differential expression patterns of the protein polo-like kinase 1 (PLK1; Fig. 2B).

We also validated the expression levels of *EPHA4* by RT–qPCR, because microarray analysis identified this as the most downregulated gene (Table 1). The *EPHA4* expression levels were dramatically suppressed in PB-22 (*P* < 0.001) and SL-22 cells (*P* = 0.020) compared with those in PB-15 and SL-15 cells, respectively (Fig. 2A). The reduced expression of EphA4 in PB-22 and SL-22 cells was also confirmed at the protein level (Fig. 2B).

The RT–qPCR results showed that expression of *KLF2* and *NOTCH2* was significantly downregulated and upregulated, respectively, in PB-22 cells (*KLF2*: *P* = 0.010; *NOTCH2*: *P* = 0.002) and SL-22 cells (*KLF2*: *P* = 0.016; *NOTCH2*: *P* = 0.018; Fig. 2A). Given these results, we next tested the *KLF2* and *NOTCH2* genes for the presence of somatic mutations. We screened the entire coding regions of *KLF2* (exons 1–3) and *NOTCH2* exon 34 in which somatic mutations are frequently reported^7, 9, 10^. Somatic mutations were not found in PB-15 and PB-22 cells and cell lines SL-15 and SL-22, except for a silent mutation, c.528C > A, in *KLF2* exon 1.

### Antiproliferative effect of PLK1 inhibition evaluated using *PLK1*-specific small hairpin RNA (shRNA) and small interfering RNA (siRNA)

**Figure 3:**
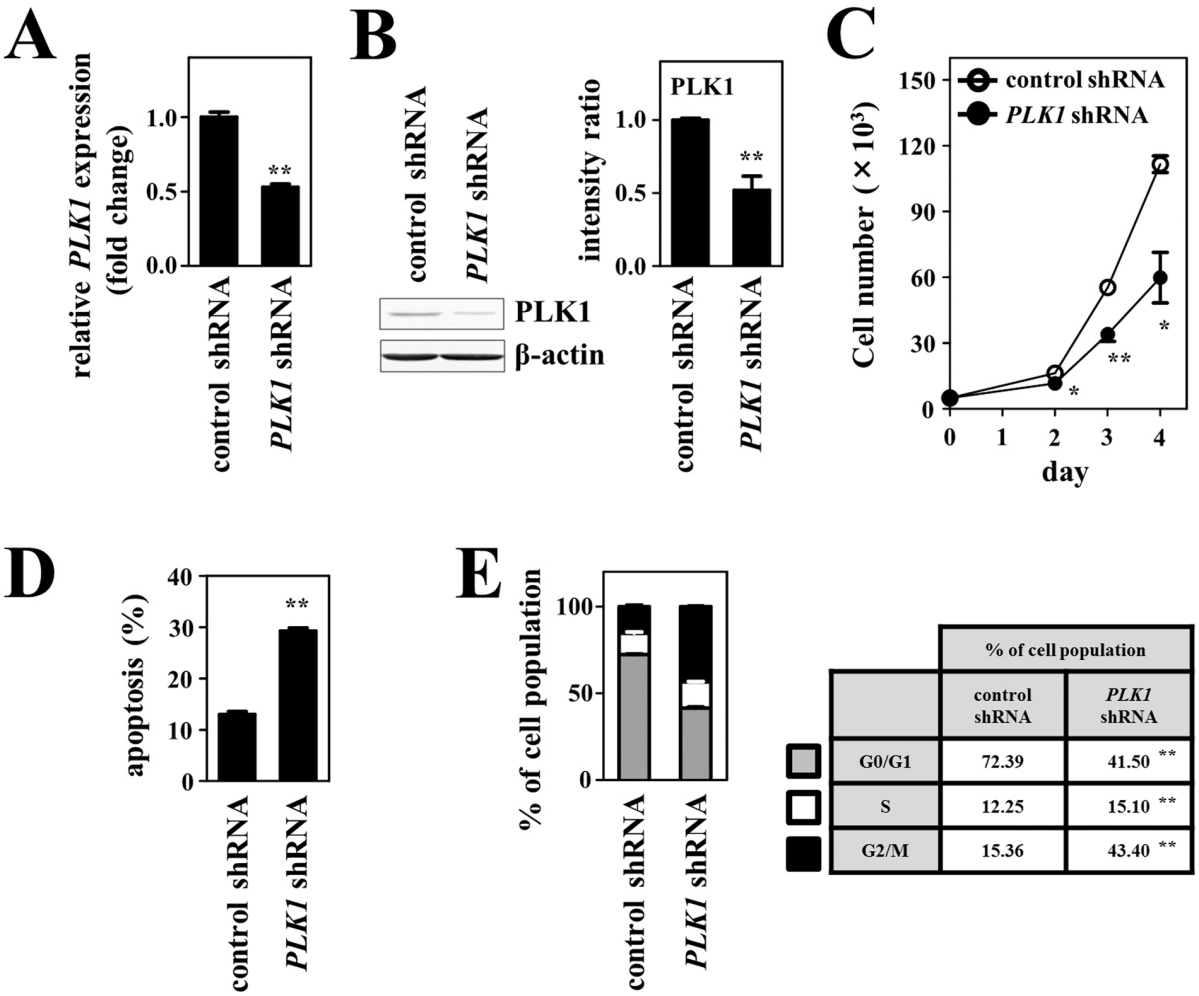
Effects of *PLK1* knockdown through shRNA on cell growth, apoptosis and the cell cycle in SL-22 cells. (**A**) Analysis of mRNA expression. The graph shows relative *PLK1* mRNA levels in cells transfected with *PLK1*-specific shRNA versus control shRNA. Expression of mRNA was assessed using RT–qPCR at 48 h after transfection. (**B**) Analysis of protein expression. The graph shows relative PLK1 protein levels in cells transfected with *PLK1*-specific shRNA versus with control shRNA. Protein levels were assessed using immunoblotting analysis at 72 h after transfection. Intensities of the bands obtained by immunoblotting were quantified and normalized to the levels of β-actin. The full-length blots are presented in Supplementary Fig. S4. Transfection of *PLK1* shRNA resulted in a significant decrease in the expression of PLK1 at both the RNA and protein levels. (**C**) Cell growth assay. After transfection of *PLK1* shRNA or control shRNA, viable cells were counted every 24 h. (**D**) Apoptosis assay. This was performed at 48 h after transfection with *PLK1* shRNA or control shRNA. The graph shows the percentage of apoptotic cells in the total cell population. (**E**) Cell cycle analysis. This was conducted at 48 h after transfection. Percentages of the cell population in each stage of the cell cycle are presented outside the graph. All experiments were repeated independently three times and data are expressed as the mean ± SEM. Significant expression differences are shown as *P < 0.05; **P < 0.01.

PLK1, belonging to the family of serine/threonine protein kinases, plays a key role in centrosome maturation, bipolar spindle formation, and cytokinesis during mitosis^19^. It is likely to be one of the key molecular candidates associated with malignant transformation of SMZL, and thus could be a potential therapeutic target. In this context, we assessed the effects of PLK1 inhibition through applying *PLK1*-specific shRNA on the proliferation and apoptosis of SL-22 cells. Transduction of this shRNA into the cells resulted in significant decreases in *PLK1* gene expression at the RNA and protein levels compared with transduction of control shRNA (Fig. 3A,B). Genetic knockdown of *PLK1* caused a significant decrease in cell proliferation and an increase in apoptosis in SL-22 cells (Fig. 3C,D). Next, we conducted experiments to determine whether the antiproliferative effects on PLK1 inhibition would be mediated through cell cycle inhibition. Inhibition of *PLK1* expression caused a significant increase in the proportion of the cell population at the G2/M phase of the cell cycle (Fig. 3E). Likewise, transduction of siRNA targeting different sequence of *PLK1* caused a significant decrease in cell proliferation through cell cycle inhibition and an increase in apoptosis in SL-22 cells compared with the control siRNA transfected cells (Supplementary Fig. S1).

### Differential sensitivity to chemical inhibition of PLK1

**Figure 4:**
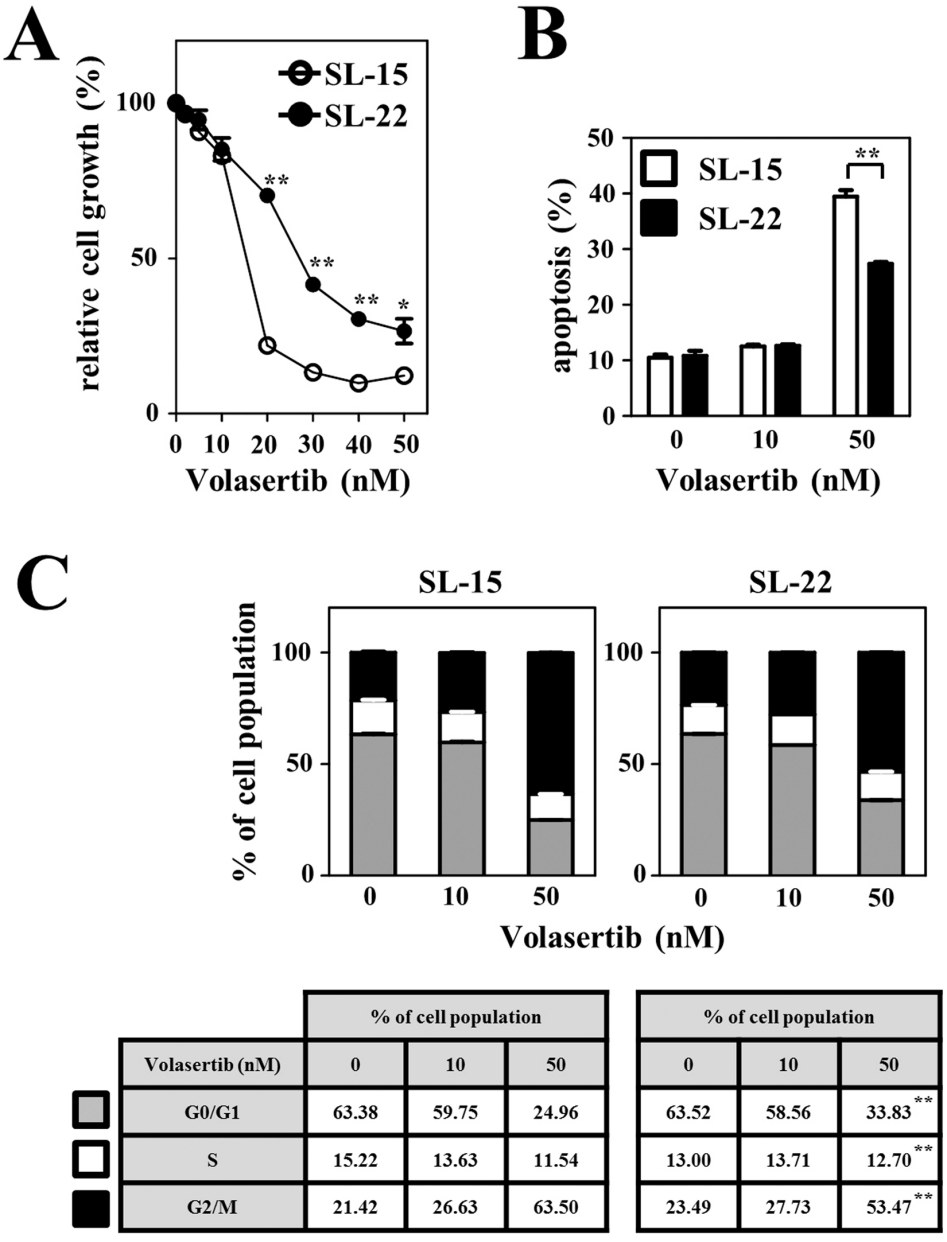
Effects of volasertib on cell proliferation, apoptosis and cell cycle. SL-15 and SL-22 cells were cultured in the presence of various concentrations of volasertib. (**A**) Cell proliferation assay. Exposure of the cells to volasertib for 48 h reduced the viable cells, but the two cell lines had diverse sensitivity to treatment with the drug. (**B**) Apoptosis assay. Cells were treated with volasertib at the indicated concentrations for 24 h. Susceptibility to apoptosis was different between the two cell lines at 50 nM. (**C**) Cell cycle analysis. After treatment of the cells with volasertib at the indicated concentrations for 24 h, cell cycle stage distributions are determined. Percentages of the cell population in each stage of the cell cycle are presented outside the graph. All experiments were independently repeated three times and data are expressed as the mean ± SEM. Significant expression differences are shown as *P < 0.05; **P < 0.01.

Next, we performed experiments to determine whether there would be a difference in the antiproliferative effect of chemical inhibition of PLK1 between SL-15 and SL-22 cell lines. Both cell lines showed no difference in cell growth in RPMI 1640 medium supplemented with 10% fetal calf serum without PLK1 inhibitors (Supplementary Fig. S2). The cells were treated with various concentration of volasertib, a selective PLK1 inhibitor, for 48 h. This resulted in efficient growth inhibition of both cell types at higher concentrations, but the two cell lines had differing sensitivities to this drug (Fig. 4A). The 50% growth inhibition (EC_50_) value of SL-22 (25 nM) was 1.9-fold higher than SL-15 (13 nM). The EC_75_ values (42 nM for SL-22 and 17 nM for SL-15) distinguished the drug sensitivity more clearly, with a 2.5-fold difference. These differences in drug sensitivity were apparent when volasertib was used at concentrations ranging from 20 to 40 nM. Similar results were obtained when another PLK1 inhibitor, BI 2536, was used (data not shown).

Exposure of SL-15 and SL-22 cells to volasertib for 24 h induced apoptosis in both lines. However, consistent with the results of the cell proliferation assay, SL-15 cells were more susceptible to apoptosis than SL-22 cells at 50 nM (*P* < 0.01; Fig. 4B). We next compared the effects of volasertib on the cell cycle between SL-15 and SL-22 cells. Although chemical inhibition of PLK1 caused an increase in the proportions of cells at the G2/M phase in a dose-dependent manner in both cells, this was significantly greater in SL-15 than in SL-22 cells at 50 nM (*P* < 0.01; Fig. 4C). Collectively, these results suggest that, compared with SL-15 cells, SL-22 cells harboring a higher expression of PLK1 needed higher levels of volasertib to be killed.

### Genomic deletion of the *MS4A1* (*CD20*) gene

**Figure 5:**
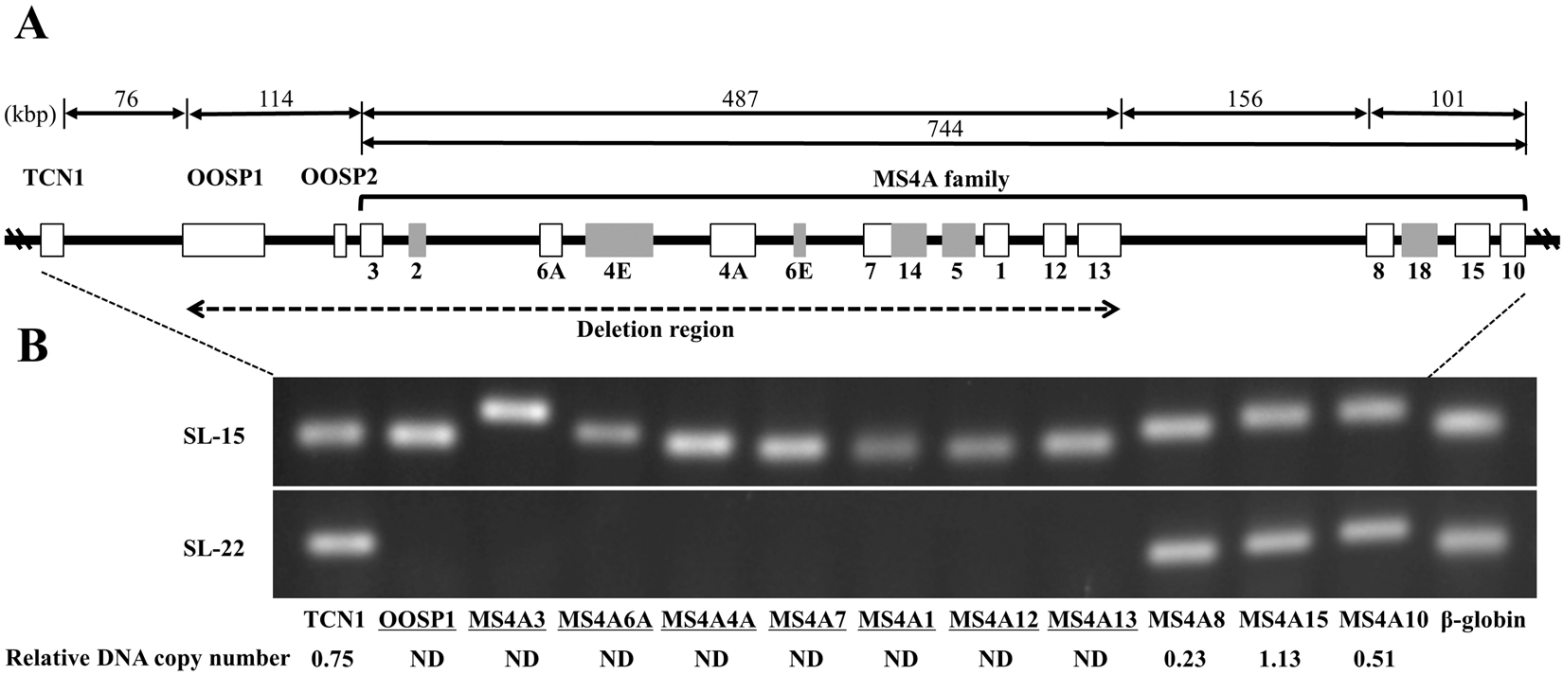
Genomic deletion of the *MS4A* family genes. (**A**) Schematic diagrams showing positions of the *MS4A* family genes (*MS4A1–MS4A18*) and their neighboring genes, including *OOSP2*, *OOSP1*, and *TCN1*. At least a 600-kb region around the *MS4A1* (*CD20*) gene has been deleted. The deletion region is indicated by a dashed line. Filled squares indicate genes that were not tested. (**B**) Results for detection of the genomic DNA by qPCR in SL-15 and SL-22 cells; the deleted genes are underlined. The numbers of copies/μg DNA in SL-22 cells were calculated and are shown as the ratio relative to that of SL-15 cells below the photographs. ND: not detected. The full-length agarose gels are presented in Supplementary Fig. S5.

PB-22 cells sampled when the tumor was refractory to rituximab-based treatment showed a CD20-negative phenotype. RT–qPCR showed that *MS4A1* mRNA was detected in PB-15 and SL-15 cells, whereas the transcript was not expressed in PB-22 or SL-22 cells. As expected, microarray analysis demonstrated over a 200-fold downregulation of *MS4A1* expression in PB-22 cells compared with PB-15 cells (Table 1). The *MS4A1* gene, located on chromosome 11q12, belongs to the *MS4A* gene family with at least 18 subgroups (*MS4A1–MS4A18*)^20^. Karyotypes of PB-22 and SL-22 cells had no chromosomal translocations and deletions involving the 11q12 region, so we suspected a partial genomic deletion around *MS4A1* in these cells. Confirming this, we identify a homozygous *MS4A1* deletion (Fig. 5). We explored the deleted span of the genomic DNA on chromosome 11q12. A 487-kilobase (kb) region from *MS4A3* to *MS4A13* within the *MS4A* cluster region was deleted. An upstream114-kb segment containing *OOSP1* and *OOSP2* showed further loss, whereas the region containing *TCN1* was preserved. Overall, at least a 600-kb region around *MS4A1* was missing on chromosome 11q12. This suggests that *MS4A1* along with its neighboring genes was lost after rituximab treatment.

## Discussion

In recent years, there have been advances in understanding the somatic gene alterations involved in SMZL. However, current knowledge about the genetic basis of SMZL is still incomplete and cannot explain the transitions in gene expression that enable progression to the aggressive form of the disease. We have established two lymphoma cell lines, SL-15 and SL-22, from a single patient with SMZL in the chronic and transformed aggressive phases, respectively. Cytogenetic studies and Southern blot analysis of Ig gene rearrangements confirmed the identical origin of the two cell lines. Although a few SMZL cell lines have been reported—one (VL51) from clinically stable SMZL^21^ and two (Karpas 1718 and UCH1) from the refractory form of the disease^22, 23^—the paired availability of our cell lines from a case of SMZL that manifested a long chronic phase before progression to an aggressive clinical course is invaluable in investigating the transformation mechanism of SMZL.

Somatic mutations affecting *KLF2* and *NOTCH2* appear to be the commonest genomic aberrations in SMZL, and studies suggest that cases with these mutations have an inferior outcome^2, 7–9^. However, the changing expression of *KLF2* and *NOTCH2* during tumor progression has not been delineated. Our findings showed that the trends toward lower expression of *KLF2* and higher expression of *NOTCH2* were linked tumor progression. We found a low frequency of somatic mutations in *KLF2* and *NOTCH2* in our samples, indicating that DNA mutations were unlikely to be responsible for the differential gene expression. Further studies are needed to confirm these findings in a large cohort.

Our microarray analysis identified specifically upregulated genes in transformed-aggressive SMZL cells compared with cells from the chronic stage. Both Functional Annotation Clustering and KEGG pathway analysis using DAVID showed that ‘cell cycle’ was the most significantly upregulated GO term. The cell cycle pathway is dominated with genes regulating cell proliferation and mitosis such as *PLK1*, *E2F2*, *MAD2L1*, *AURKB*, *CDCA5*, *CCNA2*, *CCNB1*, *CCNB2*, *CDK1*, *CDK2*, *PTTG1*, and *UBE2C*. Differences in the expression levels of all these selected genes were verified by RT–qPCR in both primary cells and their corresponding cell lines. Among these genes, *PLK1* was shown to have the largest differences in expression by microarray analysis as well as a RT–qPCR, suggesting that higher *PLK1* expression appears to be associated with a more severe grade of malignancy. Therefore, the role of PLK1 on cell proliferation and apoptosis was investigated further using the SMZL cell lines. Genetic knockdown of *PLK1* through shRNA- and siRNA-mediated RNA interference caused a reduction in cell proliferation through cell cycle inhibition and an increase in apoptosis. Although the selective PLK1 inhibitor volasertib also showed antiproliferative effects in both SL-15 and SL-22 cells, there were clear associations between the levels of *PLK1* expression and the sensitivity of cells to volasertib. SL-22 cells, which expressed higher levels of *PLK1* than SL-15 cells, needed higher concentrations of volasertib to achieve more efficient inhibition of cell proliferation and induction of apoptosis. The EC_50_ value of SL-22 shown in this study was similar to that of multiple cell lines derived from various cancer tissues, including carcinomas of the colon (HCT 116, EC_50_ = 23 nM) and lung (NCI-H460, EC_50_ = 21 nM), as shown in previous studies^24^. Thus, these data suggest that volasertib could serve as a potential therapeutic agent against *PLK1*-expressing SMZL tumors, as shown in many forms of solid cancer. Indeed, *PLK1* overexpression has been found in a variety of cancers in advanced stages, and several PLK1 inhibitors are currently in various stages of clinical trials^25^. In certain cancer types, such as invasive breast cancers and renal cell carcinomas, *PLK1* has significantly higher expression levels in late than in early stages^26^, which is in line with the results of the present study. Among hematological malignancies, *PKL1* is often overexpressed in acute myeloid leukemia^27, 28^. A few studies have suggested that high-grade non-Hodgkin’s lymphomas show a trend toward higher expression levels of PLK1 than low-grade forms^29, 30^. Our findings suggest that upregulation of *PLK1* might be involved in the biological aggressiveness of SMZL and promote its progression. In this context, although high levels of *PLK1* expression should be confirmed in more patients with advanced SMZL, clinical management with combination chemotherapy including PLK1 inhibitors is worth studying in the future.

It is notable that CD20 expression was lost in the late-stage tumor cells in our patient. Several studies have suggest that epigenetic mechanisms are linked to the loss of CD20 expression after rituximab treatment, and that the its expression can be restored by DNA methyltransferase inhibitors and histone deacetylase inhibitors^14–17^. Our study revealed a genomic deletion of the entire *MS4A1* gene along with its neighboring genes on chromosome 11q12. Based on the literature to date, Nakamaki *et al*.^31^ reported a case in which the *MS4A1* gene was deleted after rituximab-containing chemotherapy for the treatment of diffuse large B-cell lymphoma (DLBCL). Their report showed that genomic loss was observed intensively around a region including *MS4A1* and *MS4A5*, and spanned a 700-kb region involving some genes of the *MS4A* family. Consistent with that report, here we showed the genomic loss of a 600-kb segment around *MS4A1*. Loss of CD20 expression leads to the potential loss of a therapeutic target during relapse and/or disease progression of CD20-positive B-cell malignancies, and is often associated with poor prognosis for the patients at that point^14, 32^. Accordingly, clinicians and researchers should note that genomic losses around *MS4A1* are missed by conventional karyotyping, so it is necessary to recognize this genomic *MS4A1* deletion as a new molecular mechanism for CD20-negative conversion.

Our microarray identified *EPHA4* as the most downregulated gene. GO analysis revealed that *EPHA4* belonged to the category of ‘cell adhesion’ in annotation cluster 1 with the highest enrichment score. For these reasons, we payed attention to *EPHA4* as a representative downregulated gene. *EPHA4* downexpression was verified at both the RNA and protein levels by RT–qPCR and immunoblotting, respectively. Eph comprises the largest family of receptor tyrosine kinases, being composed of nine EphAs and five EphBs^33^. Recent evidence indicates that the Eph receptors have both tumor-promoting and suppressing activities, depending on their expression pattern in different tumor types; thus, some of the *EPH* genes are oncogenic and are upregulated in various cancers^33^. On the other hand, the *EPH* genes can act as tumor suppressors, and loss of their expression is evident in some tumors, for example, *EphB4* in colorectal and breast cancers^34, 35^. Likewise, EphA4 has also been found to have a multifaceted function as a tumor suppressor and promoter in some solid cancers^33, 36–38^. However, its role in the pathogenesis of hematological malignancies has not been fully determined. DNA methylation of *EPHA4* has been observed in cases of acute lymphoblastic leukemia^39^, and EphA4 expression has been shown to inhibit lymphocyte proliferation^40^. Thus, a potential role of EphA4 as a tumor suppressor in lymphoid malignancies is currently receiving increasing attention. We hypothesize that signaling pathways involved in EphA4 might be associated with the aggressive transformation of SMZL, and this should be clarified by further studies. Of note, Koivula *et al.* ^41^ showed that a low level of *EPHA4* expression was associated with poor overall survival in patients with DLBCL. Interestingly, *EPHA4* was found to be one of the most important genes associated with the responsiveness to rituximab in cases of B-cell lymphoma^41^. In this context, it is plausible that the dramatic downregulation of *EPHA4* might be caused, in part, in association with CD20 downregulation, thereby directly or indirectly contributing to the poor prognosis in our patient.

In summary, we have presented a differential gene expression profile associated with tumor progression of SMZL, and have identified specific genes for further studies to identify the molecules involved in the transformation process of this disease. Some of the gene expression changes reported here, specifically *PLK1*, might be involved in the biological aggressiveness of SMZL and could serve as potential therapeutic targets. Although a limitation of the current study was that only one case of transformed SMZL and a pair of cell lines was analyzed, future studies promise to elucidate the important issues. Another limitation of our study is the use of EBV immortalization to create the SMZL cell lines. Although their differential gene expression profile was confirmed in the primary SMZL cells, the experimental results should be interpreted with caution. If our findings are confirmed, we hope that PKL1 inhibitors will prove efficacious in improving the outcome of patients with advanced SMZL, who have limited therapeutic options. Furthermore, we have demonstrated a homozygous *MS4A1* deletion as a unique molecular mechanism of CD20-negative relapse in a patient with B-cell lymphomas. This finding suggests that cases of B-cell lymphomas with loss of CD20 should be screened for the genomic loss of *MS4A1*. Such screening will help identify patients who need early intensive treatments including stem cell transplants to overcome a CD20-negative relapse of B-cell lymphomas, because genomic deletion of *MS4A1* appears to be an irreversible event that leads to the permanent loss of the immunotherapeutic target, and ultimately to reduced survival of the patient.

## Materials and Methods

### Cell lines

The EBV-immortalized SL-15 cell line was established from a 53-year-old man with SMZL in a chronic phase. The detailed characteristics have been reported^11^. The SL-15 line was demonstrated to be derived from the clone of the patient’s primary lymphoma.

Complete remission was achieved in this patient after rituximab-based treatment, but the disease relapsed three times. After 3 years and 4 months of repeated rituximab monotherapy for each relapsed disease, the patient developed bilateral pleural effusions and ascites infiltrated with lymphoma cells. The patient died of the progressive disease with resistance to rituximab-inclusive combination chemotherapy when his white blood cell count was 60.0 × 10^9^/l with 70% lymphoma cells. The immunophenotype of these cells was similar to that of the lymphoma cells at diagnosis, with the exception that CD20 expression became negative. The karyotype of the lymphoma cells was 47, XY, add(3)(p13), add(3)(p13), t(9;14)(p13;q32), add(10)(q24), add(11)(q21), + add(11). der(11:13)(q10;q10), + 12, and add(16)(p11.2), showing a close resemblance to that of the lymphoma cells at diagnosis^11^. These findings indicated that the lymphoma cells from the pre- and post-rituximab therapy were of the same clonal origin. Following informed consent from the patient, peripheral blood was obtained 2 weeks before his death, and mononuclear cells were separated by Ficoll–Hypaque density gradient centrifugation. The cells were cultured under the same conditions used for the establishment of SL-15^11^. The cells began to proliferate after a week from initiation of the culture and could then be regularly passaged. The cell line was designated SL-22 and characterized as described^11^. This study was approved by the Ethics Committee of Kochi Medical School, Kochi University, Japan. All experiments were performed in accordance with the relevant guidelines and regulations.

### Oligonucleotide microarray

The CodeLink Human Whole Genome Bioarray (Applied Microarrays, Tempe, AZ, USA) was used to define and compare gene-expression profiles between primary SMZL cells derived from the chronic and aggressive clinical phases. The array targets most of the known and predicted genes of the human genome, and is composed of approximately 55,000 probes designated to blind to conserved exons. Labeling of complementary DNA targets, hybridization, and scanning of the arrays were carried out following the manufacturer’s instructions. Raw intensity measurements of all probe sets were background-corrected, normalized, and converted into expression measurements using the MicroArray Data Analysis Tool Version 3.2 (Filgen, Nagoya, Japan). All microarray data were submitted to Gene Expression Omnibus (https://www.ncbi.nlm.nih.gov/geo/) under accession number GSE94318. Differentially expressed genes were identified using a cutoff fold change of >2.54. GO analysis and pathway analysis (KEGG_PATHWAY) were performed using the DAVID Bioinformatics Resource 6.7 online software (https://david.ncifcrf.gov/).

### Real-time quantitative reverse-transcription polymerase chain reaction (RT–qPCR)

Real-time RT–qPCR was used to validate selected data from microarray experiments in both primary lymphoma cells and their corresponding cell lines. Total RNA was extracted using High Pure RNA Tissue kits (Roche Diagnostics, Tokyo, Japan). The total RNA was treated with DNase to avoid any amplification of genomic DNA and reverse-transcribed using the SuperScript III First-Strand Synthesis System (Life Technologies, Tokyo, Japan). An aliquot of cDNA was subjected to qPCR analysis. The reaction was conducted in triplicate on a StepOnePlus thermocycler (Life Technologies) with SYBER green PCR master mix containing 0.4 μM of each primer. The primer sequences used to determine the gene expression are listed in Supplementary Table S1. The *β-globin* (*HBB*) gene was amplified to confirm the presence of PCR-amplifiable cDNA. The PCR conditions were 10 min at 95 °C, followed by 50 cycles of 15 s at 95 °C and 1 min at 60 °C. Relative gene expression levels in PB-22 and SL-22 cells were calculated using the 2^−ΔΔCt^ method^42^, with the *β-actin* (*ACTB*) gene used as a housekeeping control, and the value was expressed as an *n*-fold change relative to that in PB-15 and SL-15 cells, respectively. Statistical analysis was performed at the ΔCt stage using unpaired two-tailed Student’s *t*-tests. A statistically significant difference was defined as *P* < 0.05.

### Immunoblot analysis

Immunoblotting was performed as described^43^. The following antibodies were used: rabbit monoclonal anti-PLK1 (clone 208G4; Cell Signaling Technology, Danvers, MA, USA); mouse monoclonal anti-EPHA4 (clone 4C8H5; Thermo Fisher Scientific, Waltham, MA, USA); mouse monoclonal anti-β-actin (clone AC-74; Merck KGaA, Darmstadt, Germany); IRDye 680RD goat anti-rabbit IgG (LI-COR Biosciences, Lincoln, NE, USA); and IRDye 800CW goat anti-mouse IgG (LI-COR Biosciences). Bands were visualized using ODYSSEY CLx (LI-COR Biosciences). Signal intensities were quantified using ImageJ software (NIH, Bethesda, MD, USA; https://imagej.nih.gov/ij/). Levels of proteins were normalized to that of β-actin.

### DNA sequencing analysis

Nested PCR was performed using Platinum SuperFi DNA polymerase (Thermo Fisher Scientific). To amplify exon 1–2 and exon 3 of *KLF2* and exon 34 of *NOTCH2*, the PCR conditions were 10 s at 98 °C (30 s for the first cycle), followed by 20 cycles of 30 s at 58 °C and 1.5 min at 72 °C (5 min for the last cycle) for the first round of PCR and 30 cycles for the second round of PCR. Amplification products were electrophoresed on 2% agarose gel and stained with ethidium bromide. Presence of somatic mutations in the *KLF2* (exon 1–3) and in the *NOTCH2* (exon 34) were investigated by Sanger sequencing as described^7, 9, 10^. The primer sequences used for nested PCR and sequencing analysis are listed in Supplementary Table S2.

### Plasmid construction and production of *PLK1*-specific shRNA and siRNA

The doxycycline-regulated and RNA polymerase II-inducible shRNA-expression plasmid vector, designated as pTRE3G1, was generated for this study. The pTRE3G1 plasmid vector contains the following constructs: reverse tetracycline activator coding region derived from pCMV-TET3G (Takara Bio Inc., Shiga, Japan); an improved variant of the copepod *Pontellina plumata* green fluorescent protein (max GFP) coding region derived from pmaxGFP (Lonza, Basel, Switzerland); the *GAPDH* promoter from nucleotides –376 to + 183 relative to the transcription start site; a tetracycline-response element promoter derived from pTRE3G (Takara Bio); 5′ and 3′ miR-155 flunking region derived from pcDNA 6.2-GW/EmGFP-miR (Thermo Fisher Scientific); and a *Luciferase* shRNA coding region derived from pSingle-tTS-Anti-Luc (Takara Bio Inc.) for control shRNA. The DNA fragments were synthesized using the GeneArt Strings system (Thermo Fisher Scientific) and cloned into SalI-PciI restriction enzyme sites of pcDNA3.1 (Thermo Fisher Scientific) using the In-Fusion HD cloning kit (Takara Bio Inc.), according to the manufacturer’s instructions.

For constructing the *PLK1* shRNA-expression plasmid vector, the DNA fragment containing a *PLK1* shRNA coding region was cloned into BamHI/SpeI sites of pTRE3G1. The *PLK1* shRNA sequence was designed using siDirect version 2.0 software (http://sidirect2.rnai.jp/). The hairpin targeting sequence was 5′–GGAUCAAGAAGAAUGAAUA–3′ in the *PLK1*-coding region (National Center for Biotechnology Information accession number NM_005030.5). Fluorescent-labeled siRNAs targeting the *PLK1* sequence 5′–CAGCCUGCAGUACAUAGAGCGUGAU–3′ and control siRNA were obtained from Nippon Gene (Toyama, Japan).

### PLK1 inhibition

For genetic inhibition of PLK1, cells were transfected with *PLK1* shRNA plasmid vector, *PLK1* siRNA, or their controls on Nucleofector (Lonza) using C solution and the D-23 program. The cells transfected with shRNA plasmid vectors were isolated 2 days after transfection by sorting maxGFP-expressing cells on a FACSAria II flow cytometer (Becton Dickinson, Mountain View, CA, USA). The isolated cells were treated with 1 μg/ml of doxycycline for 48–96 h to induce the expression of shRNA before further experiments. The transfection efficiency of siRNAs was more than 85%, as determined using fluorescent siRNA. For chemical inhibition of PLK1, cells were treated with the PLK1 inhibitor volasertib (BI 6727) (ChemScene, Monmouth Junction, NJ, USA) or BI 2536 (ChemScene) at concentrations of 5–50 ng/ml.

### Cell proliferation, apoptosis and cell cycle analyses

For cell proliferation assays, cells were seeded in 96-well plates (8 × 10^4^ cells/well) and viable cells were counted after 48 h or every 24 h on a FACSCalibur flow cytometer (Becton Dickinson) by gating out cells stained with propidium iodide as described^43^. For apoptosis assays, cells were stained with annexin V–phycoerythrin and 7-amino-actinomycin D according to the manufacturer’s instructions. For cell cycle analysis, cells were fixed in cold 70% ethanol, treated with RNase, and stained with propidium iodide. Cells were analyzed using a FACSCalibur as above, and all flow cytometry data were analyzed using CellQuest Pro software (Becton Dickinson). All experiments were performed in triplicate.

### Real-time qPCR for detecting genomic DNA of the *MS4A* gene cluster

Real-time qPCR was used to detect and quantify genomic DNA of the *MSA4* gene cluster and their neighboring genes. Genomic DNA was extracted using the phenol–chloroform method. The reaction was conducted in duplicate with 200 ng of extracted DNA and SYBR green PCR master mix containing 0.4 μM of each primer. The primer sequences used to determine the gene levels are listed in Supplementary Table S3. The PCR conditions were 10 min at 95 °C, followed by 30 cycles of 15 s at 95 °C and 1 min at 60 °C. Relative gene loads in SL-22 cells were calculated using the 2^−ΔΔCt^ method, with the *β-globin* (*HBB*) gene used as housekeeping control, and the value was expressed as an *n*-fold change relative to that in SL-15 cells. The PCR products were separated electrophoretically on 2.0% agarose gels, visualized with ethidium bromide staining, and photographed.

### Data Availability

All data generated or analyzed during this study are included in this published article and its Supplementary Information files.

## Electronic Supplementary material

Supplementary Information
