# Supplementary material for: Differential gene expression profiling linked to tumor progression of splenic marginal zone lymphoma
Source: Sci Rep. 2017 Sep 8;7:11026. doi: 10.1038/s41598-017-11389-5 (PMC5591298; doi:10.1038/s41598-017-11389-5)
Supplement: Supplementary file 1 — Supplementary Information [file 41598_2017_11389_MOESM1_ESM.pdf]

Differential gene expression profiling linked to tumor progression of splenic marginal zone lymphoma

Tomonori Higuchi[1,*], Yumiko Hashida[1,*], Ayuko Taniguchi[2], Mikio Kamioka[3] and Masanori Daibata[1]

[1] Department of Microbiology and Infection, Kochi Medical School, Kochi University, Nankoku, Kochi 783-8505, Japan

[2] Division of Hematology and Respiratory Medicine, Kochi Medical School, Kochi University, Nankoku, Kochi 783-8505, Japan

[3] Department of Laboratory Medicine, Kochi Medical School, Kochi University, Nankoku, Kochi 783-8505, Japan

*These authors contributed equally to this work.

Correspondence and requests for materials should be addressed to M.D. (daibatam@kochi-u.ac.jp)

Supplementary Table S1. Sequences of primers used for RT–qPCR.

Target	Sequence (5'→3')		Product size	Nucleotide position	Accession number
AURKB	F	GGTCCTCTTCAAGTCCCAGATAG	107 bp	440-546	NM_004217.3
	R	TGTAGAGACGCAGGATGTTGG		(exon 5- 6)	
CCNA2	F	CAGCCTGCAAACTGCAAAG	113 bp	1340-1452	NM_001237.4
	R	AAGGCAGCTCCAGCAATAAC		(exon 5- 6)	
CCNB1	F	TGTGGATGCAGAAGATGGAG	105 bp	718-822	NM_031966.3
	R	TTTGGTCTGACTGCTTGCTC		(exon 4- 5)	
CCNB2	F	CTCAAAAGCCGTCAAAGACC	116 bp	1333-1419	NM_004701.3
	R	GGCACAATGAAGCACACATC		(exon 8)	
CDCA5	F	ACCACCCGAGAAACAGAAAC	96 bp	795-890	NM_080668.3
	R	TCAAACTCGGCATTCATGG		(exon 5- 6)	
CDK1	F	TGGATTCTATCCCTCCTGGTC	88 bp	414-501	NM_001786.4
	R	TGACAAAACACAATCCCCTGTAG		(exon 4- 5)	
CDK2	F	GACCCGACTCGCTGGCGCTTCAT	115 bp	220-334	NM_001798.4
	R	GCGCCACCACCTCTCCCGTCAACTT		(exon 1)	
E2F2	F	AGGCCAAGAACAACATCCAG	108 bp	981-1088	NM_004091.3
	R	CCGTGTTCATCAGCTCCTTC		(exon 3- 4)	
EPHA4	F	TTGGCAACTGCCTATGCAAC	91 bp	880-970	NM_004438.4
	R	TCCGTGGAGAGAGCCTTGTA		(exon 3- 4)	
KLF2	F	CTTTCGGTGGCCCTGGTTTC	95 bp	685-779	NM_016270.3
	R	GGCGGCCGCGTCGTCGAAGA		(exon 2)	
MAD2L1	F	CAGTGCACCCAGAGAAAAGTC	91 bp	463-553	NM_002358.
	R	TGGCAGAAATGTCACCGTAG		(exon 3- 4)	
MEF2C	F	CCATCAGCCATTTCAACAAC	83 bp	1588-1670	NM_002397.4
	R	GCGGTGTTAAACCCAGACAG		(exon 9- 10)	
NOTCH2	F	ACACTTGCCACTGTGTGCTG	77 bp	2747-2823	NM_024408.3
	R	ACAAGGGTTTGGGGAACAG		(exon 15- 16)	
PLK1	F	CCCATCTTCTGGGTCAGCAAG	89 bp	1380-1468	NM_005030.5
	R	AAGAGCACCCCCACGCTGTT		(exon 7- 8)	
PTTG1	F	GGGACCCCTCAAACAAAAAC	94 bp	330-423	NM_004219.3
	R	ATCATCTGAGGCAGGAACAGAG		(exon 3- 4)	
UBE2C	F	TCAAATGGGTAGGGACCATC	100 bp	299-398	NM_007019.3
	R	GCATTGTAAGGGTAGCCACTG		(exon 3- 4)	
GAPDH	F	GGAAGGTGAAGGTCGGAGTCA	101 bp	193-293	NM_002046.5
	R	GTCATTGATGGCAACAATATCCACT		(exon 2- 3)	

Supplementary Table S2. Sequences of primers used for nested PCR and sequencing analysis.

Nested PCR primers

**Target**	**Sequence (5'→3')**		**Product size**	**Nucleotide position**	**Accession number, Region**
KLF2	F	AGCCACTCACCGGTGTCC	1257 bp	49-1305	NC_000019.10
(outer)	R	AAGGGAGGTCCTAAATTCATTTTC		(exon 1-exon 2)	16324826-16328662 (3836 bp)
KLF2	F	AGCCACTCACCGGTGTCC	1225 bp	49-1273	NC_000019.10
(nested)	R	GCTGGGAATCCGACCTCT		(exon 1-exon 2)	16324826-16328662 (3836 bp)
KLF2	F	AAGGCCCTGGTTAGGGATAG	510 bp	1934-2443	NC_000019.10
(outer)	R	TTGAAAAACAAAACTCGTCAAGG		(exon 3)	16324826-16328662 (3836 bp)
KLF2	F	AGGGGAACTGACGCTTACTCTC	450 bp	1994-2443	NC_000019.10
(nested)	R	TTGAAAAACAAAACTCGTCAAGG		(exon 3)	16324826-16328662 (3836 bp)
NOTCH2	F	ATTTCATCGGGGAGATAATTAGG	1583 bp	152860-154442	NC_000001.11
(outer)	R	GATGACCTTCATTTGTTCCTCAG		(exon 34)	119911553-120073449 (161896 bp)
NOTCH2	F	ATTTCATCGGGGAGATAATTAGG	1539 bp	152860-154398	NC_000001.11
(nested)	R	ATGTCTCTACACTGGAGGTGGAC		(exon 34)	119911553-120073449 (161896 bp)

Sequencing primers

**Target**	**Sequence (5'→3')**		**exon**	**Nucleotide position**	**Accession number**
KLF2	R	GCAGCCCACGTTCTACTACC	1	245	NC_000019.10
KLF2	F	TAGGGTGGTAAAAGGCAAGC	2	318	NC_000019.10
KLF2	F	CTTCGTGCATGCGAGGTC	2	785	NC_000019.10
KLF2	R	GACCTCGCATGCACGAAG	2	802	NC_000019.10
KLF2	R	CAGATGCGAACTCTTGGTGTAG	2	1182	NC_000019.10
KLF2	R	CGGGCCTTCAGTCACAGAC	3	2399	NC_000019.10
NOTCH2	F	GCCAATCGAGACATCACAGA	34	153055	NC_000001.11
NOTCH2	R	GGGAATCAACAGGGGATAAA	34	153415	NC_000001.11
NOTCH2	F	CATGAAATGCAGCCTTTGG	34	153568	NC_000001.11
NOTCH2	R	TGGATGGAGCCTACTCAAGC	34	153693	NC_000001.11
NOTCH2	F	ATCATCCTTTCCCAGCCTCT	34	154073	NC_000001.11
NOTCH2	R	TTGTGTGGTGGCTCAGACAT	34	154349	NC_000001.11

Supplementary Table S3. Sequences of primers used for qPCR.

Target	Sequence (5'→3')		Product size	Nucleotide position	Accession number, Region
TCN1	F	GAACTACTCAACCGCCGAAGT	104 bp	4963--5044	NC_000011
	R	AGGGAATTGGGCTTAGTAACTCA			59852808-59866568 (52967 bp)
OOSP1	F	AACTTAGACGAACGCCGCTGTT	106 bp	22693-22776	NC_000011
	R	AACCCAAAGAAATCCACCTCGAC			59942879-59995845 (13761 bp)
OOSP2	F	AATCTGTATATATTTGCGGATG	104 bp	3257-3360	NC_000011
	R	CCACAATCACGAACAAGA			60040275-60048044 (7770 bp)
MS4A3	F	GAGTCACCGGACCTATGCAATTAC	138 bp	10485-10599	NC_000011
	R	CAAATTATGGCACATTGGTACCTG			60056587-60071128 (14542 bp)
MS4A6A	F	TGTTCCCAATGAGACCAT	116 bp	2954-3069	NC_000011
	R	CTGCGTGTAGATGTTTCTTC			60171607..60184666 (13060 bp)
MS4A4A	F	ACTGCCCTGATGAGCCTTAG	103 bp	16672-16774	NC_000011
	R	CCCAAATTGTGTACCCGATA			60280541-60308972 (28432 bp)
MS4A7	F	GCCTCTTCCTCCTTGCTGA	101 bp	10958-11058	NC_000011
	R	GTACTCCGAATAAGGCAATG			60378485-60395954 (17470 bp)
MS4A1	F	CACTCGGAAGAGGCCATGT	94 bp	6411-6504	NC_000011
	R	TTTGGCAGTCTTACCTTG TG			60455809-60470752 (14944 bp)
MS4A12	F	GCACAAACTGTGGCTGGTCTAC	94 bp	135-205	NC_000011
	R	TTTCAACTGGCCTCAACATAGAGT			60492742-60507430 (14689 bp)
MS4A13	F	GCTGGCGTGAGAACTCGGACT	99 bp	148-246	NC_000011
	R	CAGCAAAGCGACATCACTACCCTG			60515382-60543424 (28043 bp)
MS4A8	F	ACTTCAGCAGTTCCGGTGGC	115 bp	1300-1414	NC_000011
	R	CTTGCGGCTGGCTGTTTG			60699574-60715811 (16238 bp)
MS4A15	F	CTTCCTGCCAAACGCCTTC	122 bp	18747-18868	NC_000011
	R	CAGGTGCCACATCTGCTACTCA			60756867-60776733 (19867 bp)
MS4A10	F	ATTTCAGGGATCTTGGCGATAAC	126 bp	6968-7070	NC_000011
	R	CCCTACAGACAGATGCCCTTAACA			60785304-60801305 (16002 bp)
ß-globin	F	ACACAACTGTGTTCACTAGC	110 bp	14-123	NC_000011
	R	CAACTTCATCCACGTTCACC			5225466-5227071 (1606 bp)

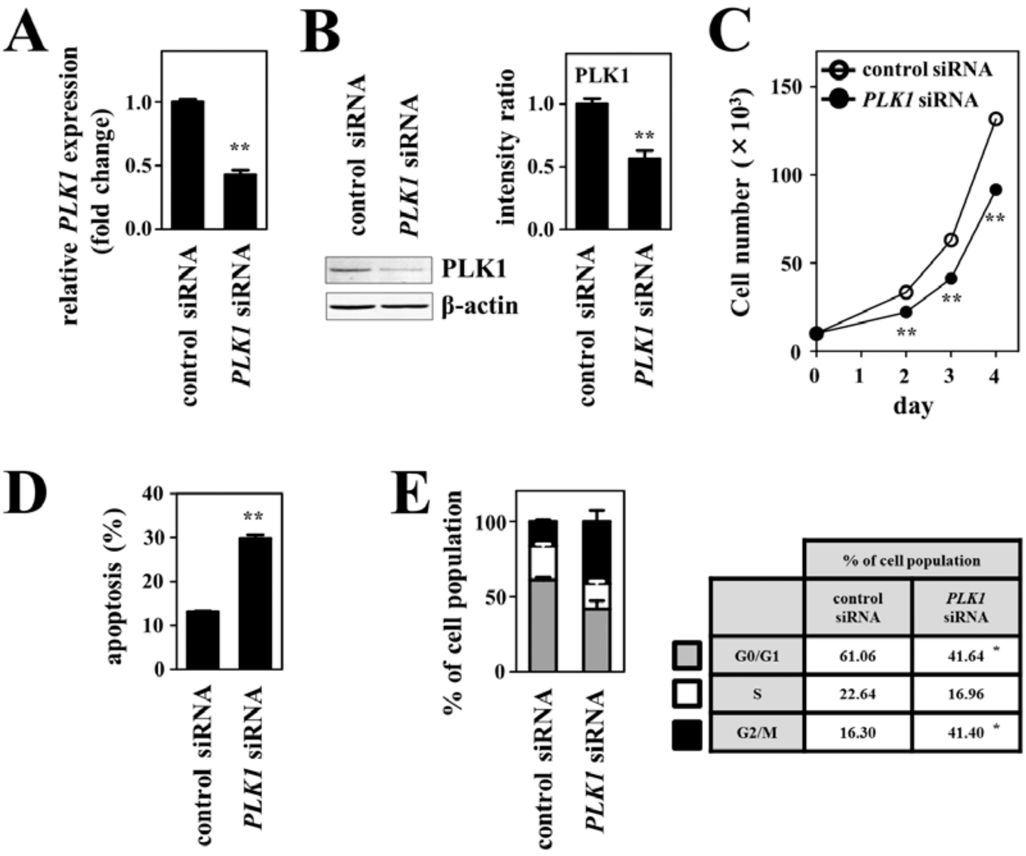

Supplementary Figure S1. Effects of *PLK1* knockdown through siRNA on cell growth, apoptosis and the cell cycle in SL-22 cells. (**A**) Analysis of mRNA expression. The graph shows relative *PLK1* mRNA levels in cells transfected with *PLK1*-specific siRNA versus control siRNA. Expression of mRNA was assessed using RT–qPCR at 48 h after transfection. (**B**) Analysis of protein expression. The graph shows relative PLK1 protein levels in cells transfected with *PLK1*-specific siRNA versus with control siRNA. Protein levels were assessed using immunoblotting analysis at 72 h after transfection. Intensities of the bands obtained by immunoblotting were quantified and normalized to the levels of β-actin. The full-length blots are presented in Supplementary Figure S6. Transfection of *PLK1* siRNA resulted in a significant decrease in the expression of PLK1 at both the RNA and protein levels. (**C**) Cell growth assay. After transfection of *PLK1* siRNA or control siRNA, viable cells were counted every 24 h. (**D**) Apoptosis assay. This was performed at 48 h after transfection with *PLK1* siRNA or control siRNA. The graph shows the percentage of apoptotic cells in the total cell population. (**E**) Cell cycle analysis. This was conducted at 48 h after transfection. Percentages of the cell population in each stage of the cell cycle are presented outside the graph. All experiments were repeated independently three times and data are expressed as the mean ± SEM. Significant expression differences are shown as *P < 0.05; **P <0.01.

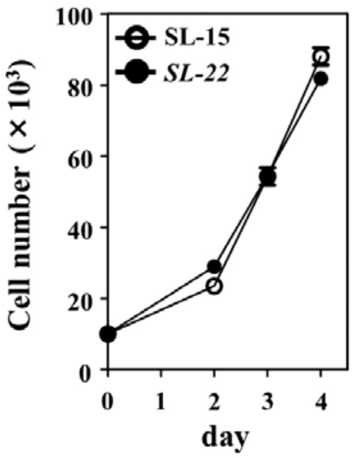

Supplementary Figure S2. Growth curves of SL-15 and SL-22 cells. SL-15 and SL-22 cells in PMI1640 medium supplemented with 10% fetal calf serum were seeded in a 96-well plate (1.0×10^4 cells/well) and viable cells were counted on FACSCalibur (Becton Dickinson) by gating out cells stained with propidium iodide.

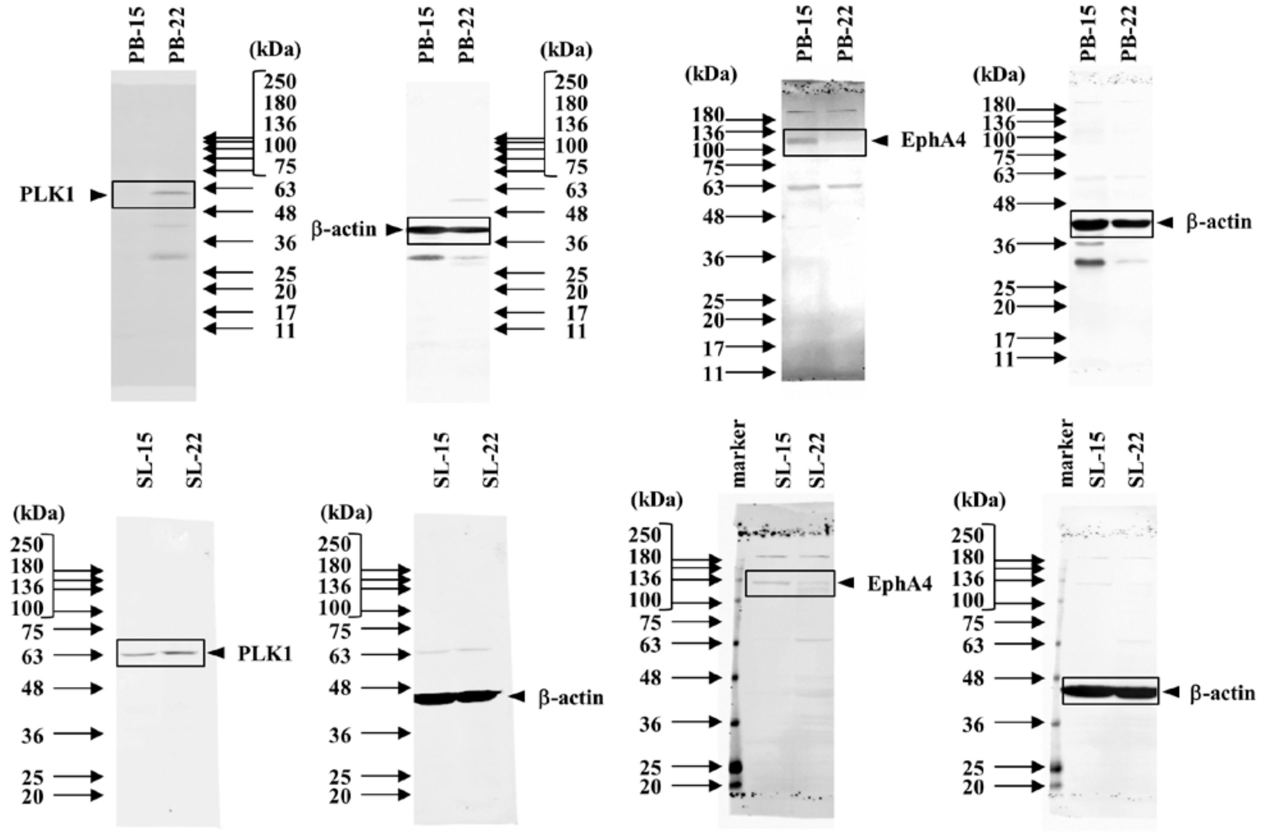

Supplementary Figure S3: Full length blots of Figure 2B. Croplines are indicated in the full length blots. After reaction with the anti-PLK antibody or the anti-EphA4 antibody, the same blot was reblocked with skim milk and then reacted with the anti-β-actin antibody.

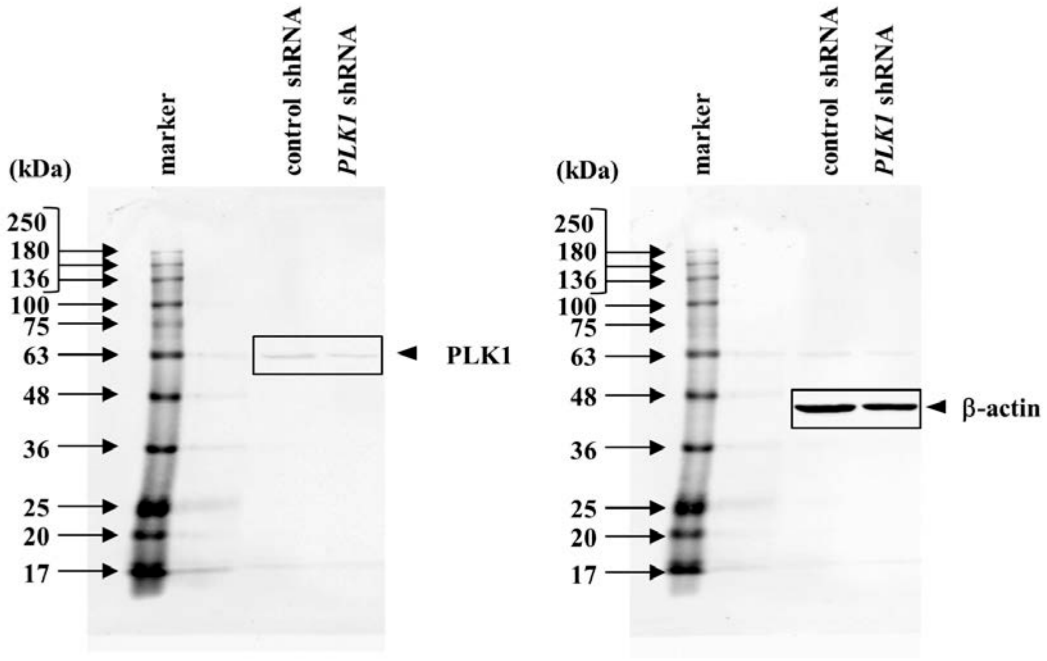

Supplementary Figure S4: Full length blots of Figure 3B. Croplines are indicated in the full length blots. The immunoblot analysis was performed on the same blots. After reaction with the anti-PLK antibody, the blot was reblocked with skim milk and then reacted with the anti-β-actin antibody.

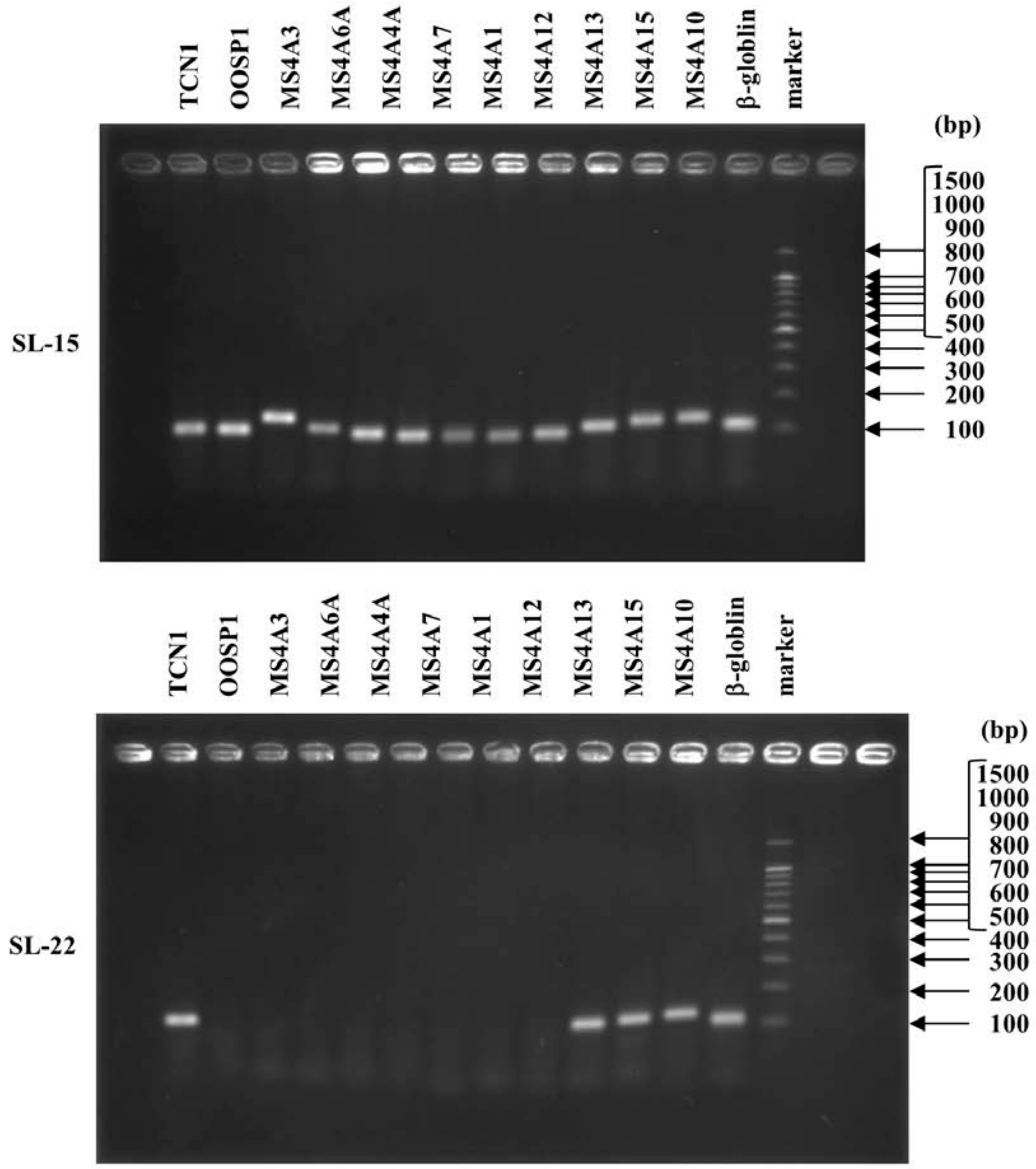

Supplementary Figure S5: Full length agarose gels of Figure 5B. Croplines are indicated in the full length gel images.

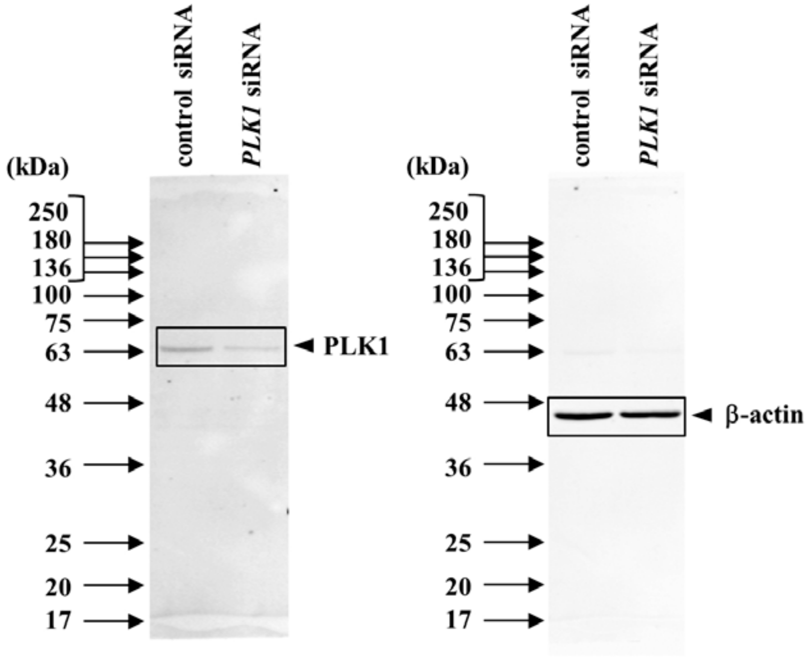

Supplementary Figure S6: Full length blots of Supplementary Figure S1B. Croplines are indicated in the full length blots. The immunoblot analysis was performed on the same blots. After reaction with the anti-PLK antibody, the blot was reblocked with skim milk and then reacted with the anti-β-actin antibody.
